# Ecohydraulogical Characteristic Index System of *Schizopygopsis younghusbandi* during Spawning Periods in the Yarlung Tsangpo River

**DOI:** 10.3390/ijerph15091949

**Published:** 2018-09-06

**Authors:** Qing-Yuan Liu, Jia Li, Rui-Dong An, Yong Li

**Affiliations:** State Key Laboratory of Hydraulics and Mountain River Engineering, Sichuan University, Chengdu 610065, China; 2016223060065@stu.scu.edu.cn (Q.-Y.L.); lijia@scu.edu.cn (J.L.); anruidong@scu.edu.cn (R.-D.A.)

**Keywords:** fish habitat protection, *Schizopygopsis younghusbandi*, statistical boundary of spawning grounds, ecohydrological indexes, ecohydraulic indexes, Yarlung Tsangpo river basin

## Abstract

To address the species decline in aboriginal fish in the Yarlung Tsangpo River Basin and the lack of research on the habitat characteristics of fish spawning grounds, this paper studied the changing trends in runoff in spawning grounds and the habitat conditions characteristics of *Schizopygopsis younghusbandi* during the spawning period. In conventional approaches, inaccurate statistical results are obtained when a full river section is taken as the region to be assessed, so a new method for determining the statistical boundaries of characteristic indexes was proposed. By combining hydrological analyses, mathematical statistics, and numerical simulations, the statistical boundary of the index was determined, and a suitable range for the habitat characteristic indexes for the spawning field was finally obtained. The results showed that (1) the maximum percentage of the statistical boundary for the spawning grounds was 39% near the banks on both sides of the river; (2) the flow during the spawning period exhibited small variations, a short duration and a fluctuation cycle and was dominated by water rising events, and the interannual growth trend in the daily flow was obvious; and (3) during the spawning period, the flow velocity of the fish habitat was small, the turbulence level of the fluid was low, and the flow regime was stable. A suitable range for the habitat characteristic index of the target fish provided the basic data for the protection of aboriginal fish and was beneficial to maintain the balance of aquatic ecological system in the Yarlung Tsangpo River. The results of this study contribute to the rational development of water resources in the basin and the protection of species diversity and water environment.

## 1. Introduction

The Yarlung Tsangpo River is one of the highest rivers in the world and possesses abundant and unique fish resources [1,2]. Its special plateau geology and climate conditions have a significant impact on the living habitats of local fish and cause the fish to show obvious regional characteristics during the long-term adaptation process. These fish have characteristics of slow growth and late sexual maturity, and they are environmentally sensitive [3]. Changes in the hydrological regime of the river easily affect these species and prevent their normal growth and reproduction. In recent years, factors such as human activity [1] and climate change have changed the hydrologic characteristics of the river. The structure and function of the ecosystem have been gradually altered, affecting the physiological activities and the physical habitat of the biological community and leading to a gradual decline in aboriginal fish resources. Although the scopes and depths of investigations have varied, fish resources in discontinuous field investigations were found to have been reduced from 43 species to 23 species from 1992 to 2007 [4,5,6,7,8]. Compared to freshwater fish in plain areas, *Schizothoracinae* have low fecundity and produce a small number of fertilized eggs. *Schizopygopsis younghusbandi*, which belongs to this subfamily, is an endemic species that produces demersal eggs and has been found only in the middle reaches of the Yarlung Zangbo River. It has been listed as a data deficient species by the World Conservation Union [9]. Most of the related studies on *S. younghusbandi* have been biological; they include a study of origin and evolution of the species and report on its food composition and development [10,11]. The habitat characteristics of these species during the spawning period have not yet been described. The spawning period is critical for the survival and reproduction of fish. Therefore, from the view of protecting population diversity and richness of each species, studying the habitat characteristics of *S. younghusbandi* in fish spawning grounds and ensuring the environmental requirements for their growth and reproduction play a positive role in restoring natural population resources.

Hydrologic situations and fluid conditions have been recognized as key environmental factors that affect the spawning and reproduction of fish [12,13,14,15,16]. Numerous methods and systems for studying and classifying the characteristics of fish spawning grounds have been proposed [17,18]. Although there are many published studies of sturgeon spawning grounds in China, related studies of *S. younghusbandi* have not yet been reported. Zhang et al. [19] used the acoustic Doppler velocimeter (ADCP, which is manufactured by American RD Instruments company) to observe the section velocity of Chinese sturgeon spawning grounds. The average measured velocity ranged from 72.99 to 175.23 cm/s. Yang et al. [20] calculated the average vorticity in a cross-section of the sturgeon spawning grounds. The minimum vorticity of the fertilized egg concentrated area was 0.4/s. The authors believed that vorticity increased the fertilization rate of demersal eggs. Based on the indicators of hydrologic alteration (IHA) [21] and environmental flow components (EFC), Zhang [22] established an ecohydrology index system for the upper reaches of the Minjiang River. He also calculated the ecological flow regime of typical hydropower stations by combining the required hydrodynamic characteristics of *Schizothorax prenanti*. Using a method for habitat simulation and taking the weighted usable area (WUA) of the whole river as the decision index, Liu et al. [23] calculated the ecological base flow during the spawning period for *Schizothorax dolichonema* and completed the calculation of the ecological flow process at the Changbo power station in the upper reaches of the Jinsha River required to meet the demand for target fish spawning. Related research abroad has primarily focused on the salmon family (e.g., *Salmo salar* and river trout). The hydraulic characteristics of Atlantic salmon and brown trout spawning grounds were summarized by Louhi et al. [24]. The reported suitable ranges of water velocity for these fish were 0.35–0.65 m/s and 0.2–0.55 m/s, respectively, and the suitable water depths were 0.2–0.5 m and 0.15–0.45 m, respectively. The characteristics of the flow velocity in the spawning field for grayling in the Pollin and Suran rivers in France were analyzed by Sempeskip and Gaudinp [25]. The results showed that the reproduction of this fish was dependent on the speed of the water. Using a 2-D finite element model which combined with habitat suitability curves developed for brown trout in the Lima River, Rui Almeida et al. [26] found that the adult brown trout tended to prefer higher velocities and greater depths in both periods than the juveniles. Pragana et al. [27] also give a simulation results for the brown trout juveniles indicated its best habitat conditions for discharges between 0.5 and 1.0 m^3^/s and the adults for 5.0 m^3^/s. Kemp et al. [28] used ecology and hydraulics to define the basic concepts of functional habitats and flow biotopes, respectively, and pointed out that functional habitats were affected by hydraulic factors in rivers.

Most existing studies have described the hydrologic and hydraulic characteristics of the spawning field in terms of flow threshold, water fluctuation characteristics, suitable water velocity range and depth. However, a few studies have included both the ecohydrologic and ecohydraulic characteristics of spawning grounds. In addition, traditional methods [19,29] have mainly used the full section of the river as the statistical scope; this makes it impossible to solve the definition of the statistical boundary for spawning grounds and affects the accuracy of the suitable range of the index. Starting from the historical survival backgrounds of indigenous fish and using the breeding space as the primary study area, this paper proposes a method for quantifying the statistical boundary of the habitat characteristic index based on the preferred habitat conditions during the spawning period. The ecohydrologic and ecohydraulic characteristics of the spawning grounds of *S. younghusbandi* were studied by means of hydrological analysis, mathematical statistics and numerical simulation. Most of the indigenous fish in Tibet are endemic species, and irreversible destruction of their habitats will lead to the extinction of these species. Understanding the ecohydrological and ecohydraulic conditions required for sensitive periods in the life cycles of these fish will help to guide the protection of spawning grounds. In addition, delving into the characteristics of ecohydrological evolution and the essential elements of life for the survival of endemic fish in the plateau area has scientific significance for the rational development of water resources in the Yarlung Tsangpo River and the diversity of aquatic river life, especially the protection of endemic fish species.

## 2. Study Area and Data

### 2.1. Study Area

**Figure 1:**
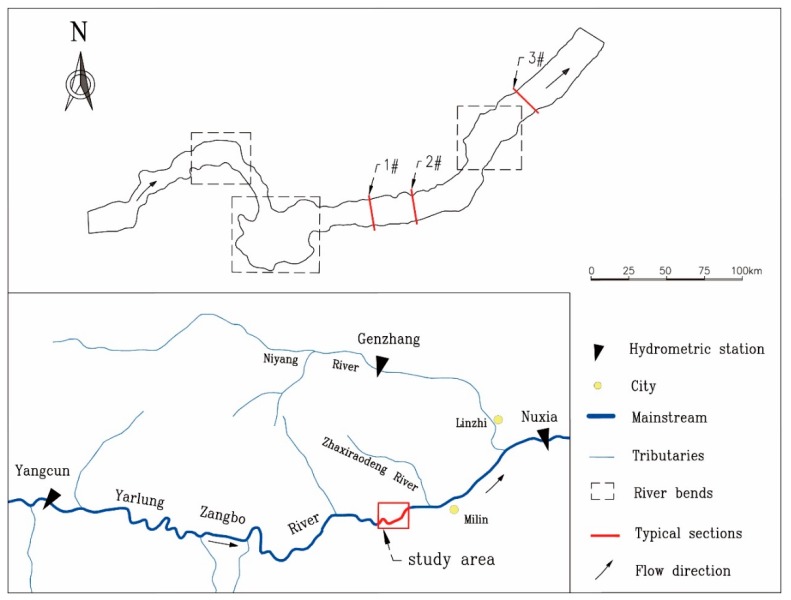
Water system in the study area.

The Yarlung Tsangpo River originates from the central glacier in Jemma, which lies west of the Tibet autonomous region and the northern foothills in the middle of the Himalayas. This river runs across southern Tibet from west to east. The area of the river basin is 2.4 × 10^4^ km^2^, and the river’s full length is 2057 km. The study area is located in the middle reaches of the Yarlung Tsangpo River (latitude 29°19′–29°20′ N, longitude 93°98′–94°04′ E and altitude 2922–2931 m). The lower reaches of the river contain two tributaries, the Zhaxiraodeng River and the Niyang River. The average slope is 1.19%, and the roughness of the river ranges from 0.02 to 0.041. The geomorphology of the river section is rich and changeable; the floodplain and terrace of the two banks are developed, and the water flow is relatively slow and steady. The topography and hydrology of the river make it a major site for *S. younghusbandi* spawning. This species is mainly distributed in the mainstream and tributaries of the middle reaches of the Yarlung Tsangpo River. This fish spawns from March to April every year and produces demersal eggs. The fertilized eggs are often deposited between rock and gravel or adhere to plants in the bottom of the river. The field aquatic survey shows that there is a spawning field in the study area. The water system in the study area is shown in Figure 1.

### 2.2. Data

The flow discharge and water temperature data used in this paper (1989–2000) were derived from measured data at the Gengzhang hydrometric station on the river’s tributaries and at the Nuxia hydrometric station, which is located downstream of the confluence. The average annual flow of the tributaries from the Zhaxiraodeng River is 28.4 m^3^/s, which accounts for only 1.3% of the average annual flow in the region and can, therefore, be neglected in the calculation. Therefore, daily flow data from the Gengzhang and Nuxia hydrometric stations were used to calculate the daily discharge of the river reach. The temperature, wind speed and relative humidity data were provided by the China Meteorological Data Service Center. When using the depth-averaged 2D hydrodynamic model and the water temperature model of MIKE software (DHI CLIENT CARE, Hørsholm, Denmark) to study the fluid characteristics and temperature field distributions of the target fish spawning field, we set the average annual discharge (401.4 m^3^/s) and water temperature (9.4 °C) in spawning period (March and April) as the boundary conditions.

## 3. Methodology

### 3.1. Analysis of the Macroecological Characteristics of the Spawning Ground

It is generally believed that river flow has a great influence on the diversity and distribution of aquatic organisms living. It determines the survival opportunities and stress thresholds of different species and places certain limitations on the survival of river organisms [30]. The fluctuation characteristics of water flow are usually consistent with the characteristics of biological life rhythms. This paper summarizes the characteristics of the hydrological environment during the spawning period and determines a suitable range for the hydrological indexes of the spawning field by analyzing the overall trend of the river flow and the characteristics of the flow fluctuation. First, the overall trends of the river flow and of the target fish during the spawning period from 1989 to 2000 were calculated by the Mann-Kendall (MK) test [31,32,33], which is effective for analyzing trends in streamflow [34,35,36,37] due to not affected by extreme values or by skewness in the data. Second, the daily water rising rate, the rising water duration, the daily water falling rate, the falling water duration and the complete water fluctuation cycle were used to construct an index system for the runoff fluctuation characteristics. Based on the mathematical statistics of the daily flow rate during the spawning period, the numerical distribution range of each index was divided into several intervals, and the corresponding frequencies (*P*) of the numerical intervals were obtained. Finally, the corresponding distribution index (*D*) of each interval was obtained by normalizing the distribution frequency of each interval.

### 3.2. Definition and Calculation of the Characteristic Indexes of River Fluctuation

(1) $$R=\left(Q_{j}-Q_{i}\right)/Q_{i}$$

**Figure 2:**
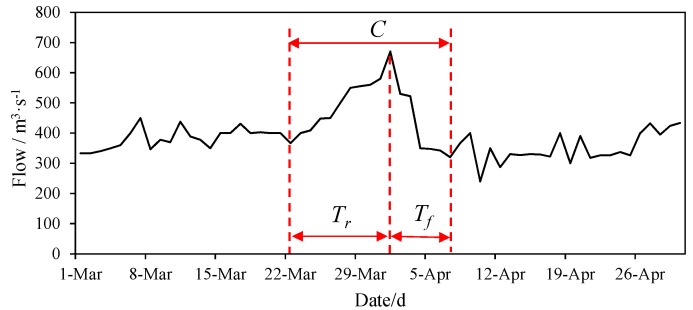
Schematic diagram of the index definition.

$Q_{i}$, and $Q_{j}$ which represent the flow discharges for two subsequent days, where *j* = *i* 1. The unit for these two discharges is m^3^/s. When *R* is greater than or equal to 0 (expressed by $R_{r}$), this formula is defined as the daily water rising rate; when *R* is less than 0 (expressed by $R_{f}$), it is the daily water falling rate. When the number of days on which $R_{r}\geq 0$, it is defined as the rising water duration (expressed by $T_{r}$), and we defined it a water rising event. When the number of days on which $R_{f}<0$, it is defined as the falling water duration (expressed by $T_{f}$), and we defined it a water falling event. The complete fluctuation cycle is defined as the time from the first flow rising event to the end of the first water falling event. The symbol for the fluctuation cycle is C, and the unit is d. A schematic diagram of the definition of each index is shown in Figure 2.

### 3.3. Analysis of the Microecological Characteristics of the Spawning Grounds

#### 3.3.1. The Determination of a Statistical Boundary

Compared with other locations in the same cross-section, *S. younghusbandi* prefers laying eggs in shoals with relatively higher temperature [9]. Thus, based on the breeding habits of the target fish, areas with large water depths should not be considered when determining the range of the characteristic indexes. The water temperature and the presence of shoals are, therefore, used as the criteria for the statistical boundary. Shoals are defined as areas in which transverse slope (α) is less than or equal to 10° or the water depth (d) is less than or equal to 0.5 m within 5 m of the ecohydraulics (α ≤ 10° or d ≤ 0.5 m within 5 m) [38]. According to the terrain features of river reach, the shoals mentioned in this paper refer to the shallow parts adjacent to the banks. Based on this definition, the percentage of the river width in the shoal is calculated, and the maximum value is taken as the reference value of the statistical boundary. The transverse distribution of the water temperature in the river is affected by factors such as topography, solar radiation, and water turbulence. Therefore, the depth-averaged 2D models of hydrodynamics and temperature are nested to calculate the river temperature field. Due to the particularity of the study area and the limitation of the data, we combined the related research findings on spawning temperature of the same species of fish (9.5–11.1 °C) [39] and the average measured temperature in the study area (9.6 °C) when determining the boundary temperature, the preferred water temperature of fish spawning under natural conditions was selected as 9.6 °C.

#### 3.3.2. The Statistics of Microscopic Characteristic Indexes

The water body of a fish spawning field has features involving spatial geometry, kinematics and dynamics [40]. The hydraulic conditions required for different fish during their spawning periods vary with the species of the fertilized egg. Studies have indicated that water depth, velocity, and the Froude number have been used to describe the hydraulic habitats of spawning grounds [41,42,43]. The water depth, as an index that describes the spatial geometry of a water body, has a significant influence on the reproduction of fish with different fertilized egg properties. The appropriate water depth can provide a suitable incubation environment for fertilized eggs, so fish will choose to spawn in such an environment. As a kinematic index, water velocity has often been considered a key factor that stimulates the spawning of fish because it increases the amount of oxygen dissolved in the water and determines whether or not the fertilized eggs of some fish hatch [44]. In addition, the Froude number, a dimensionless hydrodynamic index, can further explain how topography, flow velocity, and water depth affect fish reproduction. The Froude number is a good indicator of the structure of the fish population and the water flow pattern [45,46,47]. This paper selected three water characteristic indexes (i.e., water depth, flow velocity and Froude number) as the microecological hydrodynamic indexes of the spawning field. Other environmental factors, such as sediment and water quality, were not considered in this paper. Using the average annual flow discharge (401.4 m^3^/s) and the average annual temperature (9.4 °C) during the spawning period of the target fish as the boundary condition, MIKE 21 software was used to simulate the flow field and the temperature field of the spawning grounds, and a suitable range for each ecohydraulic index in the statistical boundary was obtained. The governing equations of the hydrodynamic model are shown in Formulas (2)–(4), while that of the water temperature model is shown in Formula (5). The hydrodynamic model is assumed to conform to the Boussinesq hypothesis, in which only the density variation caused by gravity is considered. Buoyancy is not considered in the other terms of the governing equations.

1. Continuity equation:
  (2) $$\frac{\partial h}{\partial t}+\frac{\partial h\bar{u}}{\partial x}+\frac{\partial h\bar{v}}{\partial y}=hS$$
2. Momentum equation:
  (3) $$\frac{\partial h\bar{u}}{\partial t}+\frac{\partial h{\bar{u}}^{2}}{\partial x}+\frac{\partial h\bar{uv}}{\partial y}=f\bar{v}h-gh\frac{\partial \eta }{\partial x}-\frac{h}{\rho_{0}}\frac{\partial p_{a}}{\partial x}-\frac{gh^{2}}{2\rho_{0}}\frac{\partial \rho }{\partial x}+\frac{\tau_{sx}}{\rho_{0}}-\frac{\tau_{bx}}{\rho_{0}}-\frac{1}{\rho_{0}}\left(\frac{\partial s_{xx}}{\partial x}+\frac{\partial s_{xy}}{\partial y}\right)+\frac{\partial }{\partial x}\left(hT_{xx}\right)+\frac{\partial }{\partial y}\left(hT_{xy}\right)+hu_{s}S$$
  (4) $$\frac{\partial h\bar{v}}{\partial t}+\frac{\partial h{\bar{v}}^{2}}{\partial y}+\frac{\partial h\bar{uv}}{\partial x}=f\bar{u}h-gh\frac{\partial \eta }{\partial y}-\frac{h}{\rho_{0}}\frac{\partial p_{a}}{\partial y}-\frac{gh^{2}}{2\rho_{0}}\frac{\partial \rho }{\partial y}+\frac{\tau_{sy}}{\rho_{0}}-\frac{\tau_{by}}{\rho_{0}}-\frac{1}{\rho_{0}}\left(\frac{\partial s_{yy}}{\partial y}+\frac{\partial s_{xy}}{\partial x}\right)+\frac{\partial }{\partial y}\left(hT_{xx}\right)+\frac{\partial }{\partial x}\left(hT_{xy}\right)+hv_{s}$$
3. Governing equation for water temperature:
  (5) $$\frac{\partial h\bar{T}}{\partial t}+\frac{\partial h\bar{u}\bar{T}}{\partial x}+\frac{\partial h\bar{v}\bar{T}}{\partial y}=hF_{T}+h\hat{H}+hT_{S}S$$

The variables *u* and *v* represent the depth-averaged velocities of the *x*- and y-axes, respectively; *t* represents time. The variable *h* represents the total water depth (h=η+d); η  represents the surface elevation, and *d* represents the still water depth. In addition, the terms $h\overset{-}{u}=\int_{-d}^{\eta }udz$, $h\overset{-}{v}=\int_{-d}^{\eta }vdz$, $T_{xx}=2A\frac{\partial \overset{-}{u}}{\partial x}$, $T_{xy}=A(\frac{\partial \overset{-}{u}}{\partial y}+\frac{\partial \overset{-}{v}}{\partial x})$ and $T_{yy}=2A\frac{\partial \overset{-}{v}}{\partial y}$ are used. The model is discretized by unstructured meshes. The upstream boundary adopts a flow boundary (m^3^/s), and the downstream boundary adopts a water-level boundary (m). *T* represents the depth-averaged temperature, *H* is the source term for atmospheric heat exchange, *T_s_* is the source term for water temperature, and *F_T_* is the diffusion coefficient.

#### 3.3.3. Calculation of the Distribution Frequency and Index

(6) $$P_{i}=m_{i}/M$$

(7) $$D=P_{i}/P_{max}$$

In the formula, $m_{i}$ represents the number of times the sample appears, and *M* represents the total number of samples. $P_{i}$ represents the corresponding frequency of the sample, and $P_{max}$ is the maximum frequency of these samples. *D* represents the distribution index that corresponds to the sample; the value of *D* is 1 when $P_{i}=P_{max}.$

### 3.4. Model Validation

The size of the mesh for hydrodynamic modeling are 7 m^2^~74 m^2^. To validate the models used in this study, we used cross sections to measure the current water depth and water temperature in the same study area; the cross-section numbers are shown in Figure 1. We then compared the calculated values with the measured values (Table 1). We calibrated the models by adjusting the meshes and parameters to yield simulation results close to the measured values.

As shown in Table 1, the accuracy of water depth simulation in the hydraulic model is as high as 97%. Moreover, the simulation error for water temperature in the temperature model is less than 0.1 °C compared with the measured values. The simulated flow patterns are very close to the actual situation over the entire study area, and the error produced by the simulation is within the acceptable range for raw data accuracy. This result indicates that the model essentially reflects the actual situation in the spawning grounds.

## 4. Results and Discussion

### 4.1. Analysis of Various Time Scale Trend Tests

**Figure 3:**
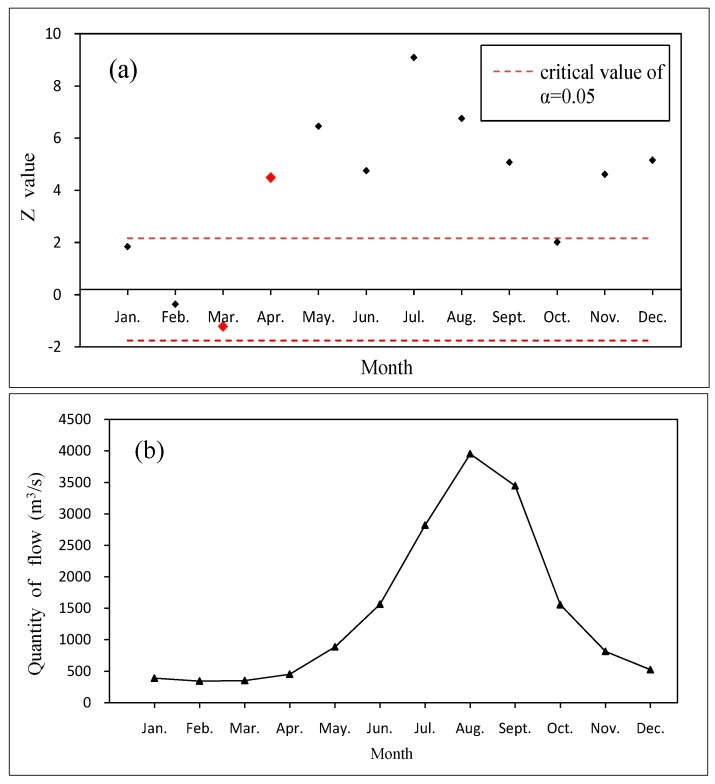
Diagram of the MK test results (**a**) and the curve for monthly average discharge (**b**).

For a given significance level of α = 0.05, the flow trend detection value was 7.6, and the confidence level reached 95%. The results showed that the daily discharge from 1989 to 2000 had a positive growth trend. Figure 3 shows the trend analysis results (a) and the curve of the monthly average discharge (b). Figure 3a shows that the trend detection value from January to March was within the standard limit. The original hypothesis of the MK trend test was established, and the flow rate was determined for a random fluctuation sample. In April, the detection value was higher than the standard limit; the flow during each month had a trend that increased each year, and the increasing trend in river flow in July was the most obvious. We can see that although the river flow exhibited an increasing trend in general, the variations in the flow during the spawning period were not exactly the same. During the secondary months of the fish spawning period, the pattern of river discharge gradually shifted from a random fluctuation to a marked increasing trend. As shown in Figure 3b, the monthly average discharge of the river first increased and then decreased, reaching a peak in August. The variation that occurred during the spawning months was not obvious, and the river discharge was relatively small during the spawning period compared with that in the other months. Following March, which showed an increase of only 3.3%, the discharge began to increase slightly when the target fish began to lay eggs. After the spawning period, the increase in river flow reached 96.2%. In summary, the natural flow of the river during the spawning period was relatively small, and the characteristics of the habitat runoff during the spawning period were not consistent. The interannual growth trend in April was more obvious when the flow was dominated by random fluctuations, and there was no obvious change trend in March. The observation that *S. younghusbandi* began to spawn when the annual flow was increasing was consistent with the idea that this species requires rising waters to stimulate spawning. 

### 4.2. Analysis of the Fluctuation in Runoff

**Figure 4:**
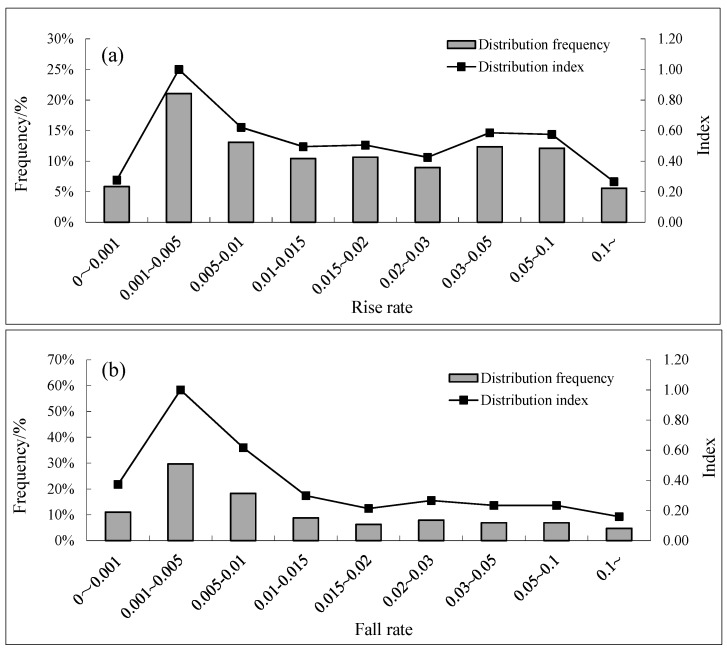
Distribution map of the rising rate (**a**) and falling rate (**b**).

**Figure 5:**
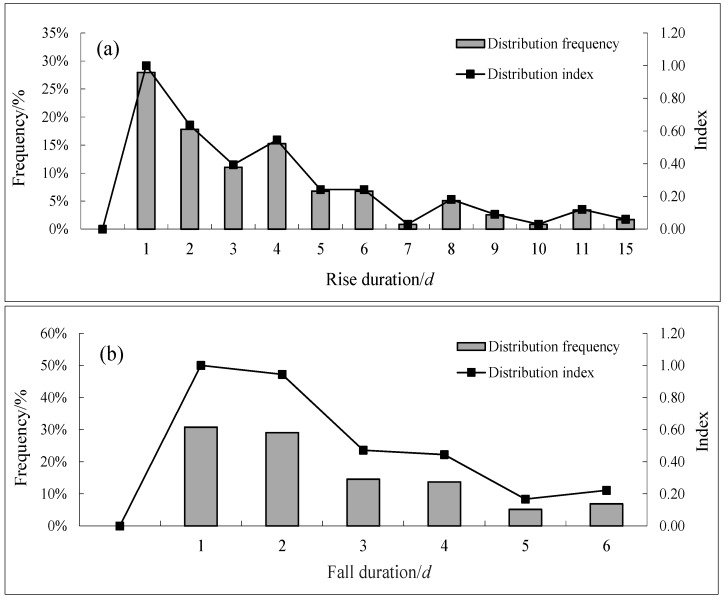
Distribution map of the rising duration (**a**) and falling duration (**b**).

**Figure 6:**
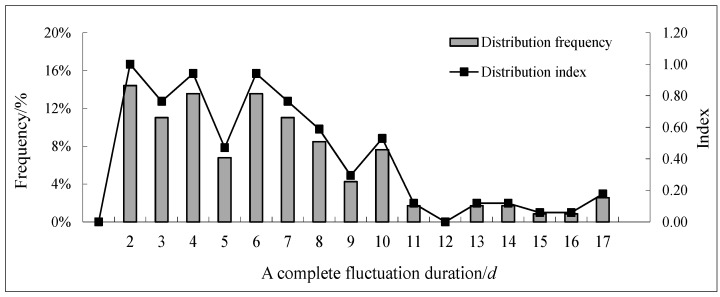
Distribution map of a complete fluctuation.

Figure 4, Figure 5 and Figure 6 show the distribution frequency of the water fluctuation characteristics for *S. younghusbandi* during the spawning period from 1989 to 2000. Based on distribution index values of 0.6 and 0.9, the water fluctuation characteristics in the spawning region are discussed. According to the data shown in Figure 4, the distribution index was greater than 0.9 when the daily water rising rate and the daily water falling rate ranged from 0.001 to 0.005; this range accounted for 21% and 30%, respectively, of the corresponding samples. Moreover, the distribution index value was between 0.6 and 0.9 when the daily water rising rate and the daily water falling rate ranged from 0.005 to 0.01. When the runoff change rate was greater than 0.01, the degree of difference in frequency decreased at each interval. Large-scale water rising and water falling events with a runoff change rate greater than 0.1 occurred 38 times, including 23 water rising events and 15 water falling incidents. Moreover, the maximum water rising rate was 0.59, and the maximum water falling rate was 0.63. In terms of the total number of occurrences of these two incidents, the number of water rising events was greater than the number of water falling events during the spawning period in the studied river. The overall water flow showed an increasing trend, and this result was consistent with the trend in the average monthly flow change rate. As shown in Figure 5a, the rising water duration was 1 day when the distribution index was greater than 0.9, accounting for 28% of the corresponding samples, and the rising water duration was 2 days when the distribution index was between 0.6 and 0.9. As shown in Figure 5b, the distribution index was greater than 0.9 when the falling water duration was between 1 and 2 days, which accounted for 31% and 29% of all corresponding samples, respectively. The maximum rising water duration was 15 days, and the maximum falling water duration was 6 days. The distribution index interval for these two durations showed a decreasing trend as the duration increased. As shown in Figure 6, the distribution index was greater than 0.9 when the complete water fluctuation cycle was 2 days, accounting for 16% of the corresponding samples, and the distribution index was between 0.6 and 0.9 when the complete water fluctuation cycle was 3, 4, 6, or 7 days; of the four cycles, 4 days showed the highest distribution frequency.

As we can see from the above discussion, the intervals were set at an ideal range when the distribution index was greater than 0.9, and the intervals were set at a suitable range when the distribution index was greater than 0.6. Therefore, the ecohydrological indicators of the target fish during the spawning period were selected. The ideal range of the water rising rate and the daily water falling rate was from 0.001 to 0.005, and the suitable range was from 0.001 to 0.01. Furthermore, the ideal range of the rising water duration and falling water duration was 1 day, and the suitable range was 1–2 days. In addition, the ideal range for the complete water fluctuation cycle during the spawning period was 2 days. The distribution index was less than 0.6 when the complete water fluctuation cycle was 5 days, which made the follow-up time and ideal range discontinuous; therefore, 2–4 days was taken as the suitable range for the complete water fluctuation cycle. With changes in the long-term natural runoff, the discharge of the river during the fish spawning period was dominated by fluctuation events with small amplitudes. However, the duration was short each time, and the frequency of large discharge changes was minimal. In terms of time distribution, water rising events alternated with falling incidents, and the duration of the two events was only 1 day. In order to further understand the habitat particularity of the hydrologic environment in the mainstream of the Yarlung Tsangpo River, we selected *Schizothorax lantsangensis* and *Schizothorax lissolabiatus* which also lay demersal eggs but mainly distributed in Lancang River (Yunnan Province) as study object. The suitable rise rate and fall rate of those species was 0–0.03 and 0.01–0.03 respectively when they are spawning [29]. The phenomenon that target fish spawn at a relatively lower frequency of rise rate and fall rate is a long-term adaptation to the environment, which may makes them more sensitive to changes in hydrological conditions.

## 5. Analysis of the Characteristics of the Flow Field in the Spawning Grounds

**Figure 7:**
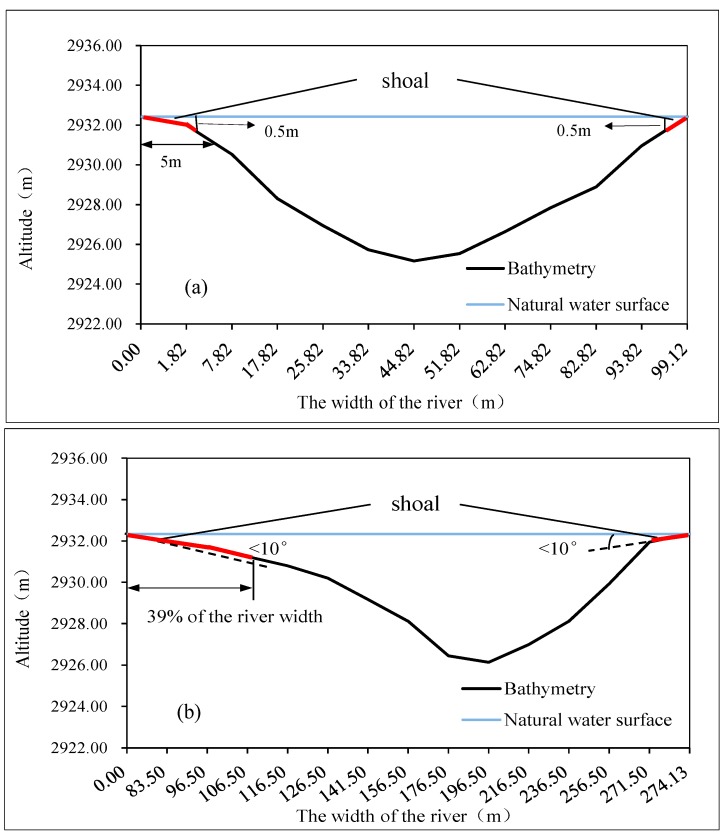
Schematic diagram of shoal boundary when d ≤ 0.5 m within 5 m (**a**) and when α ≤ 10° (**b**).

**Figure 8:**
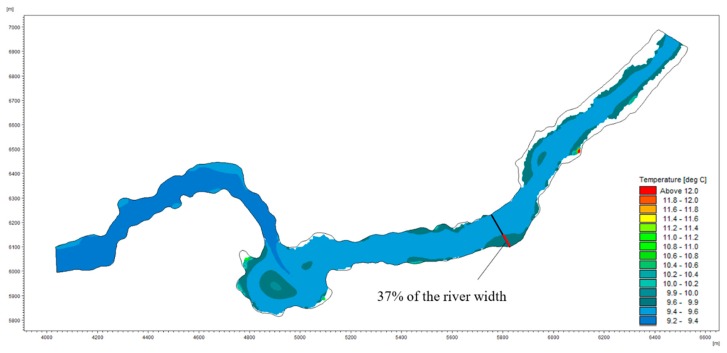
Distribution map of the water temperature field in the river section.

**Figure 9:**
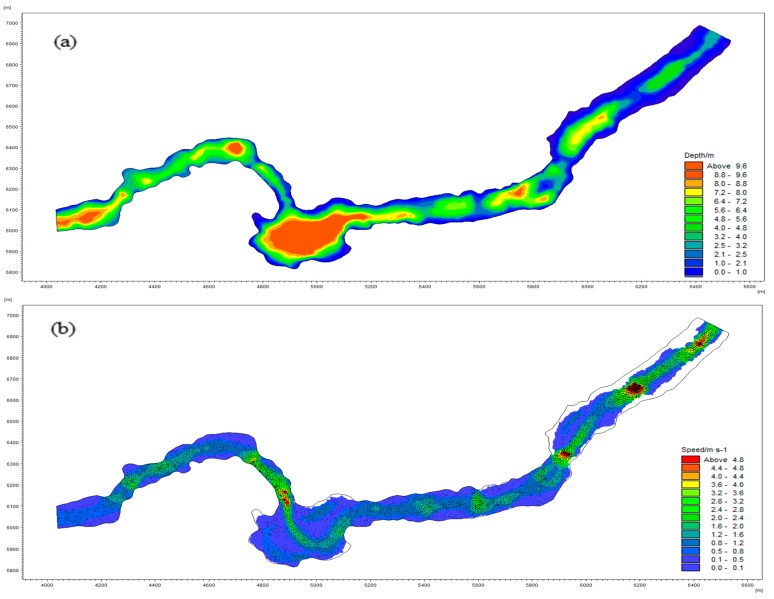
Distribution of river depth (**a**) and flow velocity (**b**).

**Figure 10:**
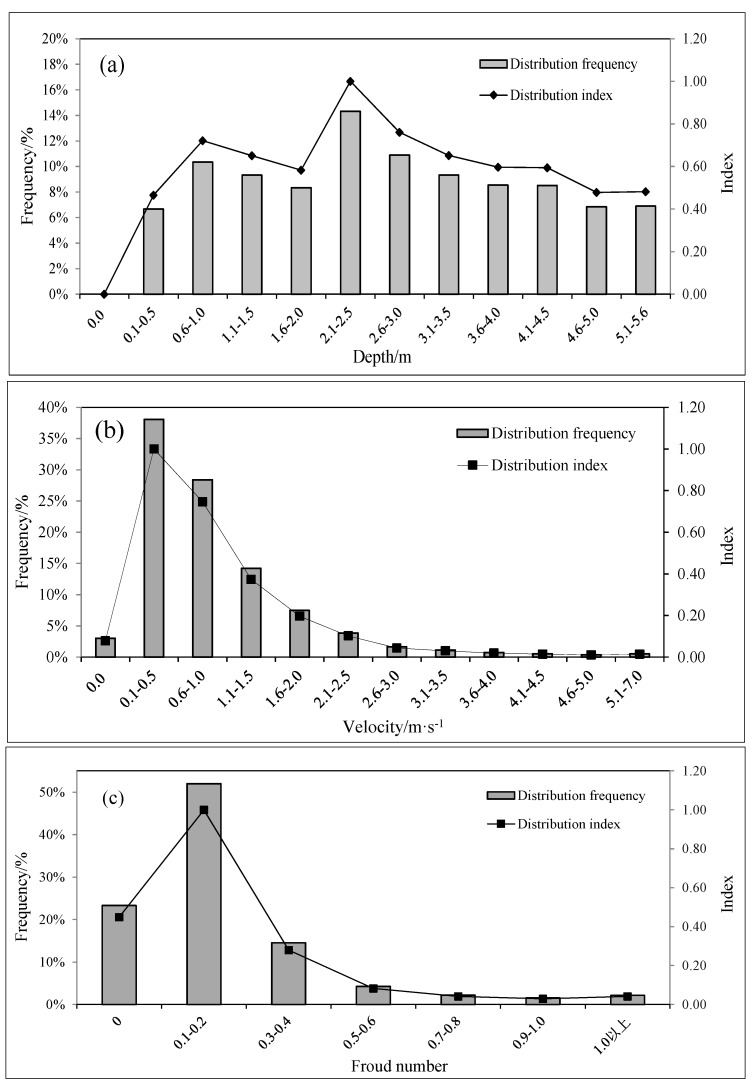
Distribution map of depth (**a**), velocity (**b**), and Froude number (**c**).

The partial analysis results for shoals are shown in Figure 7. According to the graph, the maximum shoal length is 39% of the river width. The water temperature field in the river section is shown in Figure 8. The water temperature is gradually reduced from the two sides to the center of the river, which means that the water body with a higher water temperature is mostly distributed near the banks along the sides of the river. This distribution is consistent with the habitat of the target fish when laying eggs in shoal areas. The maximum and minimum water temperatures in the river are 12.1 °C and 9.2 °C, respectively, while the distance from the boundary water temperature (9.6 °C) to the shore is 37% of the river width. According to the principle that the maximum value of the two constraints is taken as the statistical boundary, the final value of the statistical boundary is 39% of the width of the river section. The simulation results of the flow field in the river section are shown in Figure 9. The computational grid is used as a unit to extract the flow velocity, the water depth and the area of the corresponding meshes in the statistical boundary. Each data value is represented by a decimal, and the data in each index are divided into 12 equidistant intervals. The results of the numerical distributions of velocity, depth, and Froude number in the river section are obtained by calculating the distribution frequencies and the distribution indexes of the corresponding meshes in each section. A statistical analysis of the results is shown in Figure 10.

According to the flow field simulation results, the features of spatial geometry, kinematics, and dynamics in the spawning space were analyzed. Figure 9a and Figure 10a show the depth distribution map of the river section and the numerical distribution map of the water depth within the statistical boundary during the spawning period, respectively. Vertically, the depth of the upper reaches is greater than that of the lower reaches when the water depth increases horizontally from the two sides to the center of the river. The water depth is widely distributed numerically. The maximum depth of the studied river reaches 9.7 m, while that of the statistical boundary is 5.6 m. The interval whose distribution index is greater than 0.9 ranges from 2.1 to 2.5 m, accounting for 14.3% of the total grid area. When the water depth is between 0.6 and 2.0 m or between 2.6 and 4.5 m, the distribution index of each interval is between 0.6 and 0.9; when the depth ranges from 2.6 to 3.0 m, the distribution index of the interval is 0.8. From Figure 9b and Figure 10b, we can see that the degree of change in the terrain where the velocity suddenly increases is obvious. The upper reach of the river narrows at the second bend and the riverbed descends sharply, causing the velocity to increase to 2.6–5.9 m/s. After falling into wider channels, the water flow slows; the mainstream flows to the lower right side of the riverbed, and the flow direction changes from southeast to northeast. Along the near-right bank of the lower reaches, the riverbed rises due to the contraction of the river channel, and the area of low velocity again increases. Along the statistical boundary, the velocity is mainly 0.1–0.5 m/s, and the interval distribution index is greater than 0.9, accounting for 38.1% of the total grid area. In this case, the distribution interval is 0.6–1.0 m/s, accounting for 28.4% of the total grid area with a distribution index of 0.7. Generally, the flow velocity within the statistical boundary is concentrated within a range of 0.1–1.5 m/s, accounting for 80.7% of the total grid area. The Froude number (Figure 10c) for the statistical boundary of the river has a small value. Values greater than 1 occur in only a few areas, which means that the area of the torrent is very small. Most of the water bodies have slow flows, and the Froude numbers are mainly distributed in the range 0.1–0.2. Due to the change in topography and the river channel shape, the flow pattern in the area of the river near the shore is dominated by a gentle flow. This flow pattern is also consistent with the habitat of *S. younghusbandi* for laying eggs in shoals.

In accordance with the definition of the ecohydrologic characteristic index system of *S. younghusbandi* presented above, the ideal depth range of the target fish is 2.1–2.5 m, while the suitable range is 2.1–3.0 m. According to the technical guidelines [48], when the water depth is 2–3 times of the fish body length, the water space can satisfy the requirement of free swimming of fish. The average body length of *S. younghusbandi* in catch was 178 ± 98 mm and the maximum body length is 385 mm. Such a suitable range (2.1–3.0 m) can meet the requirements of free swimming, which indicates that the data obtained are reasonable to some extent. The ideal water velocity range is 0.1–0.5 m/s, and the suitable water velocity range is 0.1–1.0 m/s. Compared with *Schizothorax prenanti* (2.96 ± 0.17 mm of egg diameter [49], 1.27–2.02 m/s of velocity range in spawning ground [40] and 1.5–2.5 m of water depth [50]), the target fish (3.54 ± 0.07 mm of egg diameter, 0.1–1.0 m/s of velocity range in spawning ground and 2.1–3.0 m of water depth) has a relatively larger eggs diameter, while its suitable velocity range is smaller and suitable water depth range is larger. This velocity range may prevents the eggs from being washed away by the high velocity flow and facilitates the attachment of fertilized eggs. Such differences reflect the adaptation of fish to the environment in alpine regions, which is a combined action of appropriate hydrological conditions and water temperature. According to Duan’s [9] research, *S. younghusbandi* often lay eggs in water with a certain velocity of flow, which helps to keep the surface of the eggs clean and make it in a high dissolved oxygen environment. Therefore, even if *D* is bigger when the value is 0, we choose 0.3–0.4 as the suitable interval and select 0.1–0.4 as the suitable range of the Froude number. In summary, the ranges of the ecohydrologic and ecohydraulic characteristic indexes for target fish spawning grounds are shown in Table 2.

## 6. Conclusions

In this paper, a method for determining the statistical boundaries of spawning grounds for target fish was established. A suitable range for the habitat characteristic indexes of *S. younghusbandi* during the spawning period was obtained by hydrological analysis, mathematical statistics, and numerical simulation. The conclusions are as follows:

1. Combined with the ecological habitats of fish, a statistical boundary was calculated with shoal and water temperature as the constraints. The maximum restriction value of these two constraints was selected as the statistical boundary of the spawning grounds. This method divided the river habitat into two parts: one was the main channel habitat, and the other was the shoal habitat. These parts were used to quantify the statistical boundary of the spawning ground of the target fish to reduce the scope of statistics and improve index accuracy. In this paper, the statistical boundary, which accounted for 39% of the width of the river section in the spawning ground, was calculated. This result was consistent with the actual position of the fertilized eggs found in the field investigation.
2. The ecohydrological index system for spawning grounds of target fish was determined by five parameters in three dimensions (i.e., the rising water rate, the falling water rate, the rising water duration, the falling water duration and the complete water fluctuation cycle). The suitable range of the rising water rate and falling water rate was from 0.001 to 0.01, and the suitable range of the rising water duration and falling water duration was 1 to 2 days; the suitable range of the complete water fluctuation cycle was 2 to 4 days. Compared with *Schizothorax lantsangensis* and *Schizothorax lissolabiatus*, which also lay demersal eggs, but mainly distributed in Lancang River. The suitable rise rate and fall rate of target fish are smaller. The phenomenon that target fish spawn at a relatively lower frequency of rise rate and fall rate is a long-term adaptation to the environment, which may makes them more sensitive to changes in hydrological conditions.
3. The ecohydraulic index system for target fish during the spawning period was determined by selecting the water depth, velocity and Froude number, which utilized the forms of spatial geometry, kinematics and dynamics. The water flow was mainly a slow and steady flow in the studied river reach, and the velocity was low. The suitable velocity range was from 0.1 to 1.0 m^3^/s; the suitable range for the water depth was from 2.1 to 3.0 m, and the suitable range of the Froude number was from 0.1 to 0.4 in the spawning grounds. Compared with *Schizothorax prenanti* [50], the suitable range of velocity of target fish is smaller while the suitable depth range is larger. This velocity range may prevent the eggs from being washed away by the high velocity flow and facilitates the attachment of fertilized eggs. Besides, a larger depth can provide more adequate living space, which also helps to avoid predators. Such differences reflect the adaptation of fish to the environment in alpine regions, which is a combined action of appropriate hydrological conditions and water temperature.

During the study period, human activity had little influence on the ecological structure and function of the river; therefore, the index system can be used for restoring and constructing required hydrologic and hydraulic conditions of spawning habitat. The research data provide a reference for judging the magnitude of the change in fish habitat and basic data for protecting species diversity. However, due to the sensitivity and particularity of the study area and the lack of basic data and related research results, the precision of the index range in this paper needs to be improved. Therefore, in subsequent research, we should conduct model experiments to further study the suitable range of indigenous fish.

## Figures and Tables

**Table 1:** Results of the model validation.

Indicators	1#	2#	3#
Measured water depth/m	7.27	7.02	6.20
Simulated water depth/m	7.28	6.99	6.19
Measured water temperature/°C	9.45	9.45	9.46
Simulated water temperature/°C	9.42	9.43	9.45

Notes: 1#, 2#, 3# represent the cross-section numbers which can see in the Figure 1.

**Table 2:** Habitat characteristic index ranges for *S. younghusbandi* spawning grounds.

	Eco-Hydrological Indexes					Eco-Hydraulic Indexes		
Range	Rising Rate	Falling Rate	Rising Water Duration/Day	Falling Water Duration/Day	Complete Fluctuation Cycle/Day	Velocity/m·s^−1^	Depth/m	Froude Number
Ideal range	0.001~0.005	0.001~0.005	1	1	2	0.1~0.5	2.1~2.5	0.1~0.2
Suitable range	0.001~0.01	0.001~0.01	1~2	1~2	2~4	0.1~1.0	2.1~3.0	0.1~0.4
